# 

**Published:** 1983-10

**Authors:** 


					Centenary Year
1883-1983
k
| I
BRISTOL
MEDICO
CHIRURGICAL
JOURNAL
OCTOBER 1983
VOLUME 98 (iv)
NUMBER 368

				

